# Stone Anvil Damage by Wild Bearded Capuchins (*Sapajus libidinosus*) during Pounding Tool Use: A Field Experiment

**DOI:** 10.1371/journal.pone.0111273

**Published:** 2014-11-05

**Authors:** Michael Haslam, Raphael Moura Cardoso, Elisabetta Visalberghi, Dorothy Fragaszy

**Affiliations:** 1 Research Laboratory for Archaeology and the History of Art, University of Oxford, Oxford, United Kingdom; 2 Institute of Psychology, University of São Paulo, São Paulo, Brazil; 3 Istituto di Scienze e Tecnologie della Cognizione, Consiglio Nazionale delle Ricerche, Roma, Italy; 4 Department of Psychology, University of Georgia, Athens, Georgia, United States of America; University of Florence, Italy

## Abstract

We recorded the damage that wild bearded capuchin monkeys (*Sapajus libidinosus*) caused to a sandstone anvil during pounding stone tool use, in an experimental setting. The anvil was undamaged when set up at the Fazenda Boa Vista (FBV) field laboratory in Piauí, Brazil, and subsequently the monkeys indirectly created a series of pits and destroyed the anvil surface by cracking palm nuts on it. We measured the size and rate of pit formation, and recorded when adult and immature monkeys removed loose material from the anvil surface. We found that new pits were formed with approximately every 10 nuts cracked, (corresponding to an average of 38 strikes with a stone tool), and that adult males were the primary initiators of new pit positions on the anvil. Whole nuts were preferentially placed within pits for cracking, and partially-broken nuts outside the established pits. Visible anvil damage was rapid, occurring within a day of the anvil's introduction to the field laboratory. Destruction of the anvil through use has continued for three years since the experiment, resulting in both a pitted surface and a surrounding archaeological debris field that replicate features seen at natural FBV anvils.

## Introduction

Stone tool use is currently known to be habitual or customary among members of three wild non-human primate species: western chimpanzees (*Pan troglodytes verus*) in West Africa, Burmese long-tailed macaques (*Macaca fascicularis aurea*) in Thailand, and bearded capuchin monkeys (*Sapajus libidinosus*) in Brazil [1]–[3]. All three species use hand-held stones as pounding tools to access embedded food, and stone surfaces (including cobbles, boulders and outcrops) are among the natural substrates used as anvils by each species to support the pounded item. The use of stone for percussive tasks means that both hammers and anvils survive for a considerable period of time and can be used repeatedly, and the forceful impact associated with percussive strikes can damage the stones through fracture and abrasion. One result is the formation of pits in the surface of both hammers and anvils, which have been noted as an indicator of pounding tool use for both non-human primates and hominins [4]–[10]. Stone anvil fracture has also been posited as a potential path to the creation of sharp-edged tools through intentional stone flaking, a trait that appears confined to the hominin lineage [11], [12].

Anvil damage or use-wear is one of the primary means by which an anvil stone may be distinguished from other naturally occurring stones and outcrops [e.g.], [ 13], [14]–[16]. In order to interpret anvil damage correctly, however, we must first understand the process by which it occurs. In instances where anvil use has not been directly observed and recorded, basic questions such as the duration and intensity of past use can only be addressed through analysis of damage patterns. To help answer such questions, we present here an experimental study of use-wear formation on a stone anvil used by wild bearded capuchins, at the Fazenda Boa Vista (FBV) site in Brazil.

### Fazenda Boa Vista

FBV is located in the southern Parnaíba Basin (S 09° 39′ 49.6″, W 45° 25′ 22.5″) in Piauí, Brazil. Details of the local environment are provided in [7]. The climate is seasonally dry, with 1,290 mm of annual rainfall, and 25 mm rainfall during the dry season, May to September [17].

Capuchins at FBV use a variety of stone materials as hammers to crack open resistant palm nuts (89% of tool use episodes), as well as other encased foods [17]. Stone hammers range in weight from hundreds of grams to more than two kilograms [7], with adult individuals producing a maximum kinetic energy of 7–12 J per strike in one experiment [18]. Stone tool use occurs at a median rate of about one episode per 10 h for each tool user, accounting for around 1% of the total time budget for the group [17]. Both stone and wood anvils are used at FBV, and most of them are located in the transition zone between large sandstone mesas and flat open woodland [7], [19]. FBV stone anvils are formed from relatively soft sandstones and siltstones, which shear from the mesas and can present large, almost horizontal surfaces that average around 1.9 m^2^ [7]. Stone anvils used by capuchins around the mesa that includes the experimental area have an average Rx rebound value of 29.6, with anvil hardness across the study area reflecting the hardness of the prevailing sedimentary rock [7].

Almost all stone anvils at FBV are pitted, as a result of repeated abrasion and compression forces when foods are placed on their surface and forcibly pounded. Most anvils have fewer than 10 pits, although anvils with up to several dozen pits are present, and the maximum number of pits recorded for one anvil to date is 83 [7; MH unreported data]. Anvils on the north and east sides of the mesas are used more frequently at FBV than those with southerly or westerly aspects [16].

## Methods

**Figure 1:**
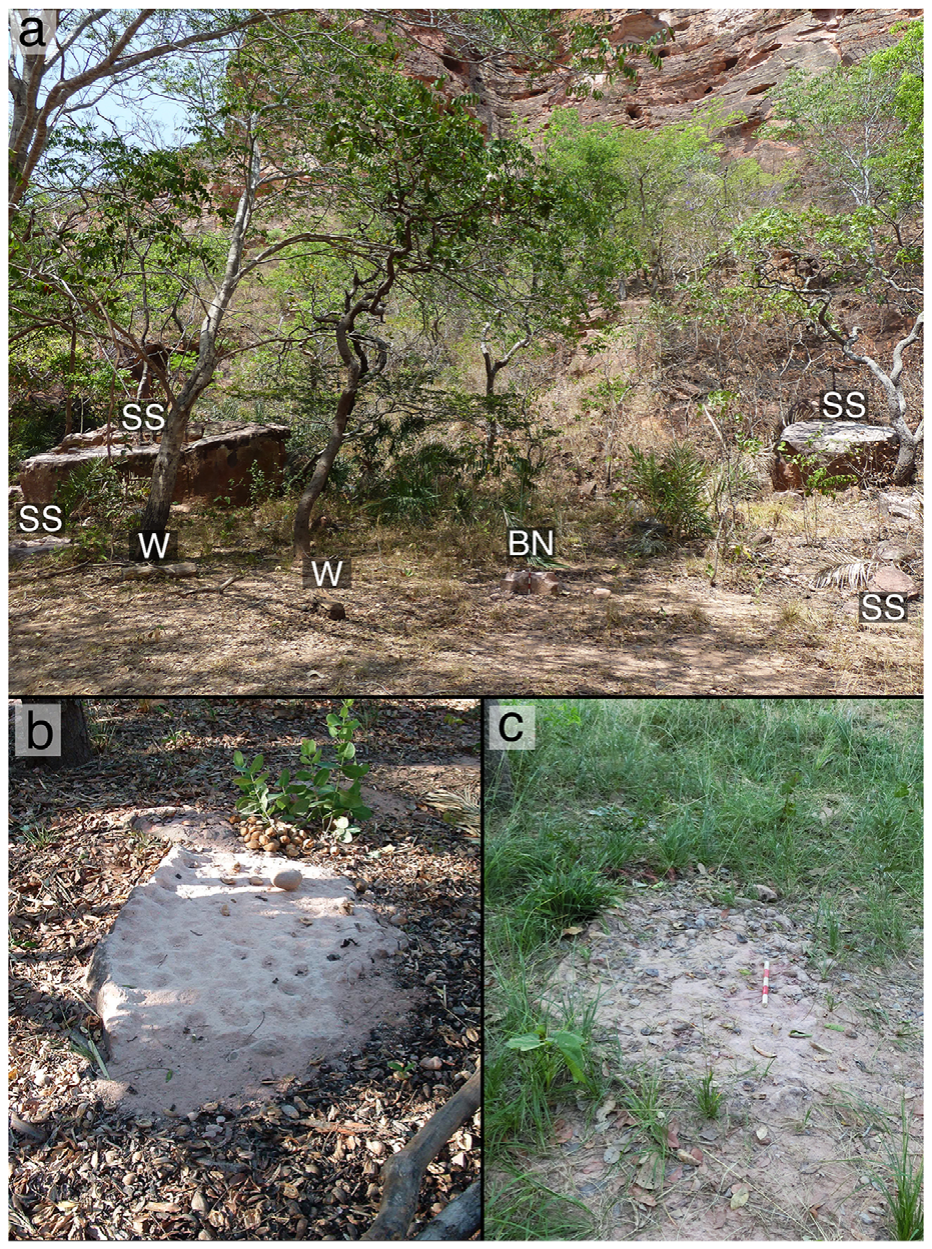
The FBV field laboratory. (a) The experimental area beside a steep mesa, with sandstone (SS) and wood (W) anvils, and the Bigorna Nova (BN). (b) A sandstone anvil at the field laboratory in 2003, and (c) the same anvil in 2014, showing the erosive effect of capuchin pounding.

Our experiment was conducted in May 2011 with the one group of wild bearded capuchins [20], which at the time consisted of 19 individuals: 3 adult males, 5 adult females, 5 immature males and 6 immature females (Table 1). The capuchins are habituated to human presence, and the experiment took place in part of the monkeys' natural range that had previously been used for other experiments [e.g.], [ 21],[22]–[24]. This ‘field laboratory’ is a flat area approximately 15 m in diameter with good visibility and several stone and wood anvils, as well as a variety of quartzite and siltstone hammers (Figure 1a). The site is located on the north-east edge of the nearest sandstone mesa.

**Table 1:** Age, sex and body mass of monkeys in the studied group, May 2011.

Individual	Age	Sex	Mass (kg)*
Piaçava	Adult	F	1.98
Teninha	Adult	F	2.18
Chuchu	Adult	F	1.96
Amaralinha	Adult	F	1.63
Dita	Adult	F	2.09
Mansinho	Adult	M	3.30
Teimoso	Adult	M	3.34
Jatoba	Adult	M	3.84
Tomate	Immature	M	1.80
Catu	Immature	M	1.81
Congaceiro	Immature	M	1.83
Pati	Immature	M	1.68
Coco	Immature	M	1.14
Doree	Immature	F	1.37
Pamonha	Immature	F	1.23
Paçoca	Immature	F	1.18
Chani	Infant	F	0.46
Thais	Infant	F	0.42
Presente	Infant	F	0.24

* Body mass was obtained using a voluntary weighing system described in [26].

**Figure 2:**
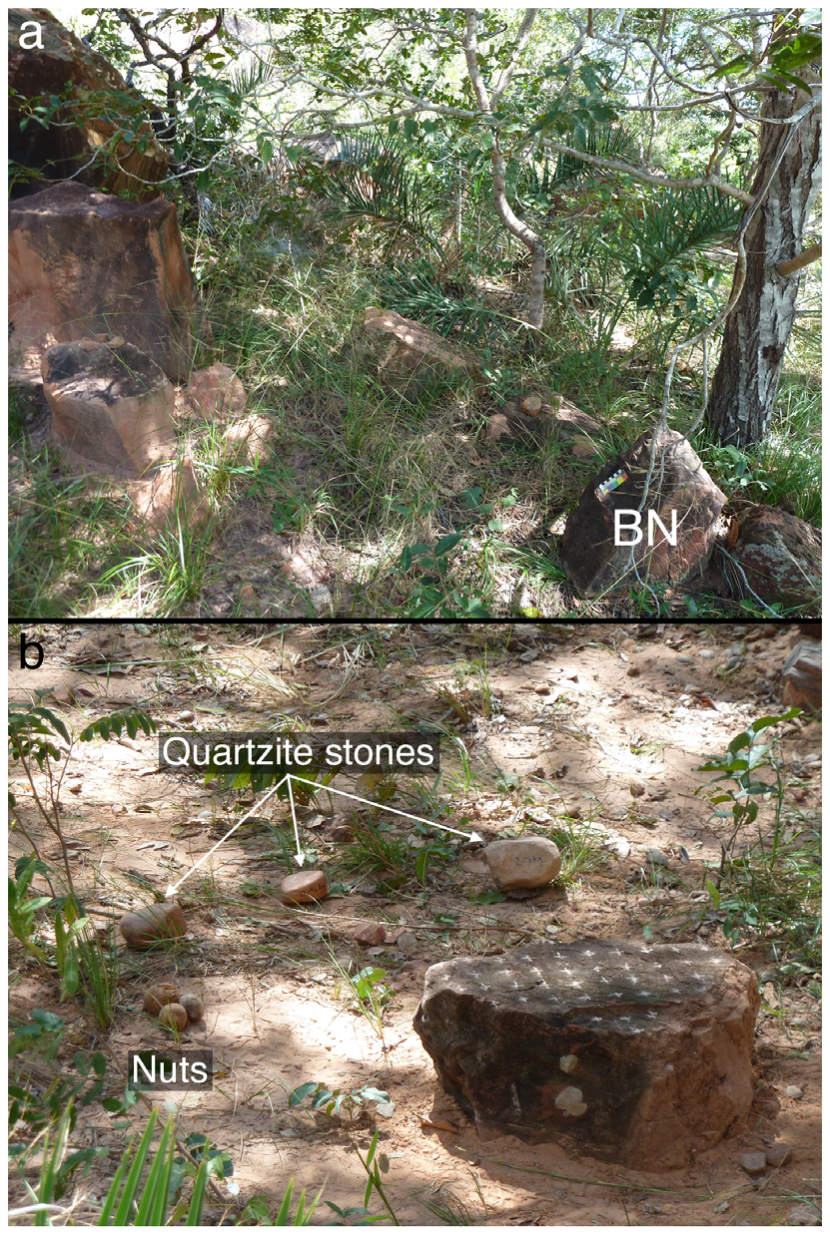
Setting up the Bigorna Nova (BN). (a) The original location and position of the stone SE of the field laboratory, with a 10 cm scale on the tilted face; (b) BN in position at the start of the experiment; the upper surface has chalked crosses every 5 cm to aid in determining strike positions.

To investigate the entire use-wear process, we set up an unused sandstone block at the field laboratory for use as an anvil (Figure 2). For ease of reference, the block was designated ‘Bigorna Nova’ (BN, literally new anvil). This block had a minimally-weathered, flat, undamaged fracture plane measuring 49×31 cm, which was found close to vertical and therefore had not been previously used by the capuchins. The fracture plane formed the horizontal upper surface of the anvil once we transferred the block to the field laboratory, which was 29 m to the north-west of the block's original location (Figure 2a). Once stabilized and leveled at its new location the BN upper surface was 24–25 cm above the slightly uneven ground, and the base of the anvil was 58×40 cm. The BN sandstone was the same colour and composition of other anvils at the field laboratory and the surrounding area.

**Figure 3:**
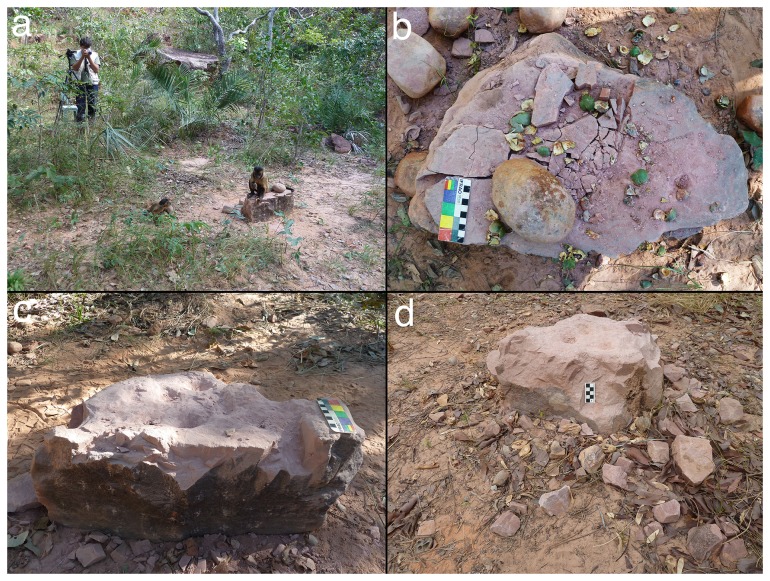
The Bigorna Nova experiment. (a) Bigorna Nova (BN) experiment in progress, with RMC operating the video camera; (b) BN surface damage during the experiment, with tucum shells and quartzite hammer stone, scale is 10 cm; (c) BN at the end of the period of continuous monitoring, note the pitted surface and surrounding debris, scale is 10 cm; (d) BN 16 months after the experiment, surrounding stone and nut debris is extensive, scale is 5 cm.

We recorded all interactions between the monkeys and BN for four days, covering the anvil with a tarpaulin to prevent capuchin access while the researchers were absent. All monkeys' activities with BN were recorded using a digital video camera set up 4.45 m from the anvil, as well as *ad libitum* digital photography (Figure 3). Three quartzite potential hammer stones were initially provided next to the anvil, weighing 0.46 kg, 1.05 kg and 2.08 kg, along with two species of nuts collected from wild plants at FBV: piaçava (*Orbignya* sp.) and tucum (*Astrocaryum campestre*). The former nut is much harder and bigger than the latter [25]. Soon after the experiment began, we restricted hammer use at BN to the 1.05 kg stone, to better control this variable. The capuchins were free to use the stones and nuts provided, or to bring additional nuts to the anvil, and they could approach and use the anvil from all sides. Additional palm nuts were provided to facilitate use of BN, and other anvils and hammers were always available in the field laboratory away from the BN study.

Each time that the monkeys used a stone hammer to strike a nut they had placed on the BN anvil, we used the video record to note the position of that strike on the anvil upper surface. At a minimum of every 20 strikes, which usually involved multiple nuts being cracked, we recorded the presence of macroscopically-observed pits in the anvil surface. We recorded pit location, and maximum dimensions of length, width (perpendicular to length) and depth. We recorded the latter by placing a plasticine ball within the pit, then laying a ruler across the top of the pit to compress the plasticine, and measuring the resulting thickness with calipers. For each strike, we also recorded the individual monkey, the nut type, and whether the nut was whole or partially broken (where this was visible on the video, or mentioned in the video by the experimenter).

We also recorded whether or not the monkey removed loose material from an existing pit (making the accessible part of the pit deeper), and/or swept loose anvil and nut debris from the surface of the anvil onto the surrounding ground. Both behaviors were labeled cleaning actions. Cleaning removed fragments of the anvil, including pieces from mm to cm in size, that had detached from the main anvil body but had remained *in situ* either within or around surface pits (Fig. 3). Previous observations indicate that this material would otherwise form a barrier between the nut and anvil, and its removal accelerates pit formation and overall damage by exposing the underlying anvil surface.

Following one week of complete monitoring, BN was left uncovered and monkey use was no longer continually recorded. For the subsequent three years on an annual basis we recorded the anvil cross section in two perpendicular planes to assess overall changes in height and surface shape, and photographed the anvil.

To provide controlled comparative data, MH created use-wear pits in a separate sandstone anvil by repeatedly dropping a 1.05 kg quartzite hammer onto positioned piaçava and tucum nuts. The hammer weight was chosen based on the reported average weight of hammers at Boa Vista of 1.096 kg [7]. The hammer stone was dropped from a height of 33 cm each time; this value was based on averaged data for maximum vertical hammer height during nut-cracking by four Boa Vista capuchins [18]. Capuchins may add force to each downward strike, which was absent in this experiment to err on the conservative side. Each drop fell squarely on the nut, and was counted as a strike, with the dimensions (width, length, depth) of the resulting pits measured every 10 strikes up to a total of 100 strikes. The anvil used in the stone drop experiment was collected from close to the BN original location. A further aim of this study was to assess variation in pit formation between tucum and piaçava that may allow for discrimination of these nuts via pit data from anvils at FBV. Specifically, we hypothesized that the rounded tucum would produce pits with a greater depth, relative to pit length and width, than the broader piaçava nuts. Pit measurements were taken as for the BN.

We calculated the odds ratio to assess whether whole or partial nuts were preferentially placed within pits.

### Ethics statement

Permission to work in Brazil was granted to EV and DF by Instituto Brasileiro do Meio Ambiente e dos Recursos Naturais Renováveis (IBAMA) and Conselho Nacional de Desenvolvimento Científico e Tecnológico (CNPq). The study was conducted on private land, owned by the family of Marino Gomes Oliveira. This research was approved by the IACUC of the University of Georgia (A2010 04-067 and A2013 03-001) and complied with all institutional guidelines for the ethical participation of non-human animals in research.

## Results

During the period of continual observation, a total of six adults (3 males, 3 females) used tools on the BN anvil, along with a number of immature individuals (Table 2). The latter were not identified to individual, and were analysed collectively. We recorded strikes on 67 tucum (n = 320 strikes) and 161 piaçava nuts (n = 479 strikes), for a total of 799 strikes. Note that these values do not necessarily reflect successful nut-cracking, and so should not be considered measures of efficiency. The capuchins created 14 identifiable pits during this period, with 579 strikes located within pits and the remainder on other parts of the anvil surface.

**Table 2:** Summary data for the BN experiment.

Individual	Strikes	Nuts	% Cleaning*	# pits started
Jatoba	169	49	39.0	9
Mansinho	119	32	36.6	1
Teimoso	159	36	4.9	3
Piaçava	130	39	4.9	1
Dita	50	14	7.3	0
Chuchu	73	4	4.9	0
Immature	99	54	2.4	0

*% of all cleaning events that were performed by this individual.

The capuchins performed 341 strikes in which the nut was placed into a pit and for which the whole or partial nature of the nut could be ascertained; 261 of these strikes were on whole nuts, and 80 on partial nuts. Of the 134 strikes in which the nut was placed elsewhere than in a pit, and for which we could ascertain whether the nut was whole or partial, 21 were on whole nuts, and 113 on partial nuts. We calculated the odds ratio of a whole nut being placed in a pit as 17.5 (χ^2^ p<0.001), indicating that monkeys preferentially place whole nuts in pits, and partial nuts outside these depressions.

**Figure 4:**
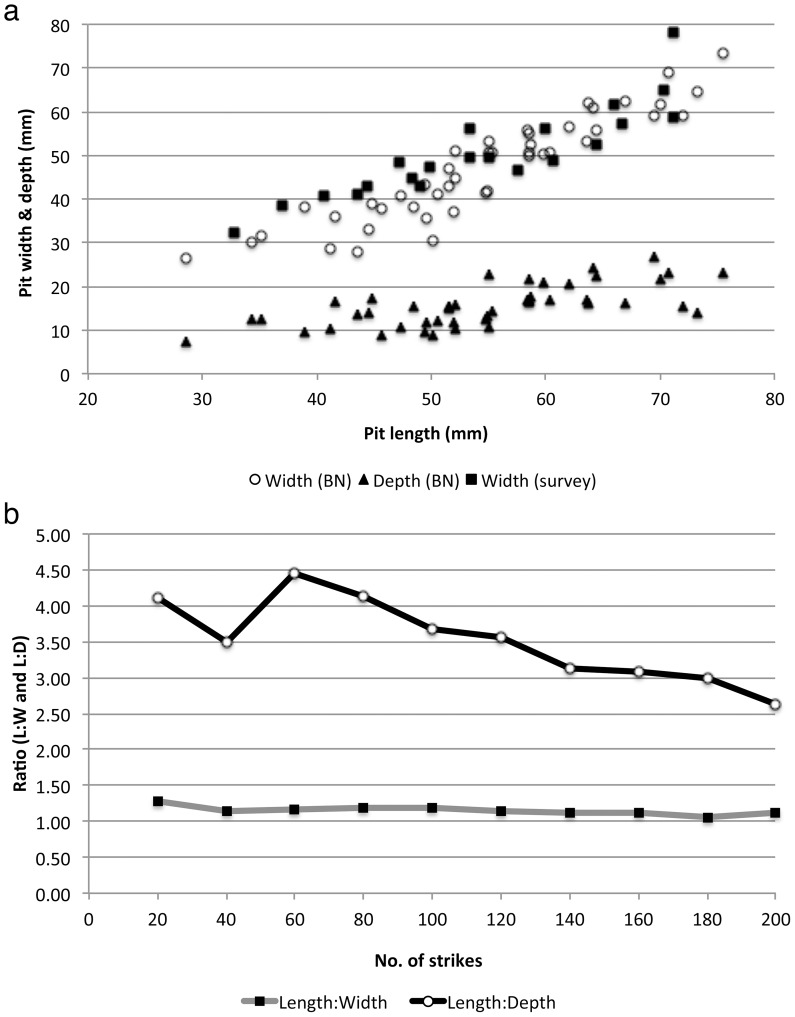
BN pit measurements, with anvil survey data for comparison: (a) BN pit width and depth relative to length, and surveyed anvil pit widths relative to length; (b) ratios of pit length∶width and length∶depth formed on the BN surface during the experiment.

Pit size data (Figure 4) indicate that pit width increases linearly as length increases (r^2^ = 0.857), while depth also increases but at a slower rate (Figure 4a). Pit depths reach a plateau typically less than 25 mm, which reflects the fact that beyond that depth the monkeys often strike the surrounding stone surface rather than the nut, resulting in the abrasion and local fracture of the anvil surface. On occasion, this can actually result in pit depth decreasing slightly as the other dimensions increase, even though the overall trend is towards an increase in all three dimensions. Because of this process, the ratio of length to depth decreases over time (Figure 4b), producing larger but shallower pits. For comparison, and to ensure that data from BN reflected natural occurrences, we also measured a sample of pits on FBV anvils that were surveyed over several years and reported in [16] (Figure 4a). The surveyed anvil pits showed similar sizes to the BN data, although they tend to be slightly wider (by a few mm) for pit lengths below 50 mm.

Most of the cleaning behavior was performed by adult male monkeys (33 cleaning events from 117 nuts) (Table 2). Adult females cleaned the anvil seven times (from 57 nuts) and immature individuals once (from 51 nuts). The current and former alpha males (Jatoba and Mansinho, respectively) were most active in this process, collectively cleaning the anvil on 38% of their visits. From these data, the main agents for accelerating anvil damage through cleaning are dominant males. Adult females play a minor role, and immature individuals very rarely engage in cleaning behavior. Monkeys cleaned almost twice as often when cracking tucum rather than piaçava nuts (27% to 14%).

Initiation of a new pit occurred when a monkey placed a nut outside of the already established pits, and cracked nuts repeatedly in that location until a macroscopically visible pit formed. Of the 14 pits created during the experiment, nine were initiated by the alpha male (Jatoba), one by the former alpha male (Mansinho), three by another subordinate male (Teimoso), and one by the alpha female (Piaçava) (Table 2). Immature monkeys never started a new pit. On average, a new pit was initiated after 10 nuts were cracked, corresponding to an average of 38.5 strikes per new pit. We also looked at whether individuals would preferentially re-use the pit that had been used by the previous monkey at the anvil, and found that the capuchins re-used the same pit 40% of the time, with similar frequency seen in this behavior between immature monkeys, adult females and adult males (44%, 46% and 37% respectively; total n = 144 events).

**Figure 5:**
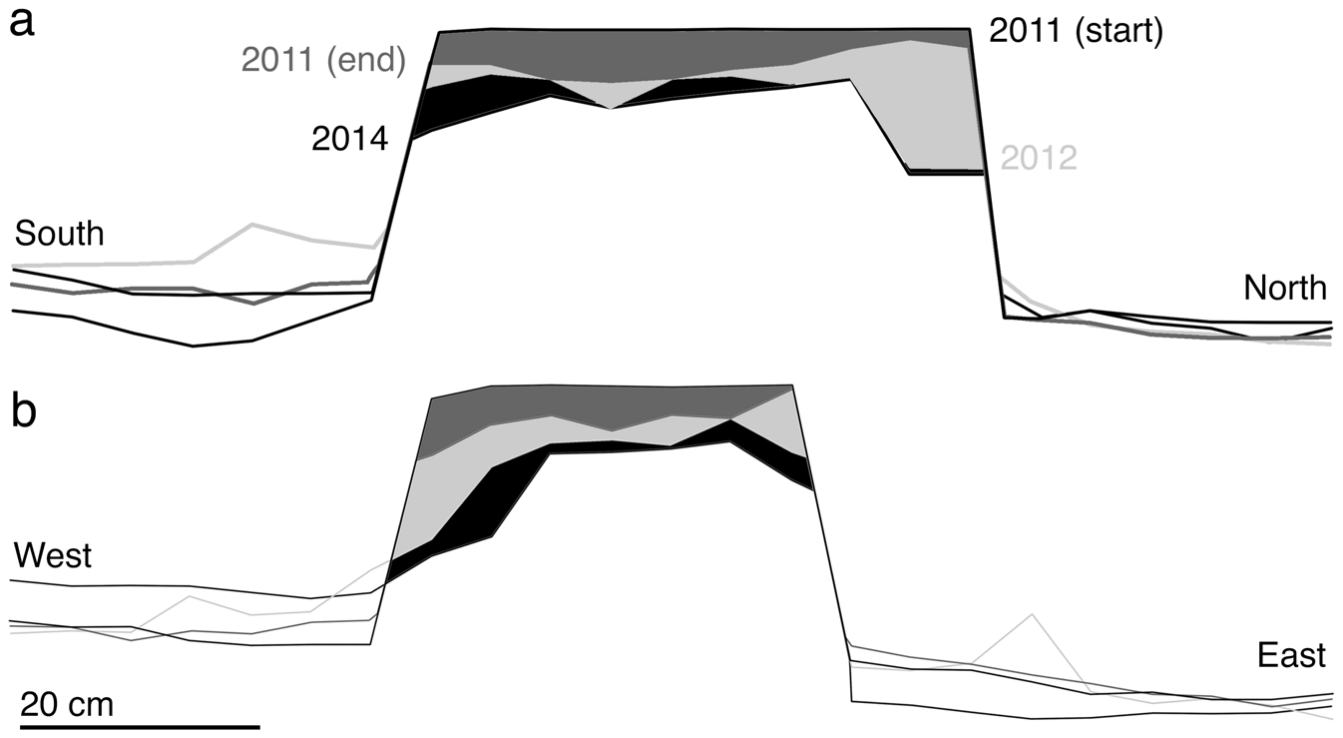
Schematic BN cross-sections, 2011–2014: (a) South to North; and (b) West to East. The uppermost level in each cross-section is the anvil shape at the start of the experiment (2011 start) and subsequent levels were taken at the end of the initial experimental period (2011 end), in September 2012, and in May 2014.

From May 2011 to May 2014, the BN anvil continued to be used opportunistically by the FBV capuchins (Figures 3d, 5). Through pounding activities, its height decreased relative to the original anvil surface by at minimum of 2.6 cm, and a maximum of 13 cm (i.e., more than half the original anvil height). The erosion of the anvil upper portion left a considerable debris field in the immediate vicinity, chiefly within 50 cm of the anvil base. During the initial one-week period of observation, the anvil decreased in height by 0.4–4.8 cm. This was 41.7% of the total mass lost, as estimated by averaging loss at points taken 5 cm apart in two cross-sections, indicating that capuchin use of the anvil during that period was considerably more intensive than in the subsequent three years. We suggest that the latter period is more representative of normal use patterns, although the change in intensity affects only the overall rate of wear, not the mechanisms involved. The anvil upper surface remained pitted throughout the three-year period.

**Figure 6:**
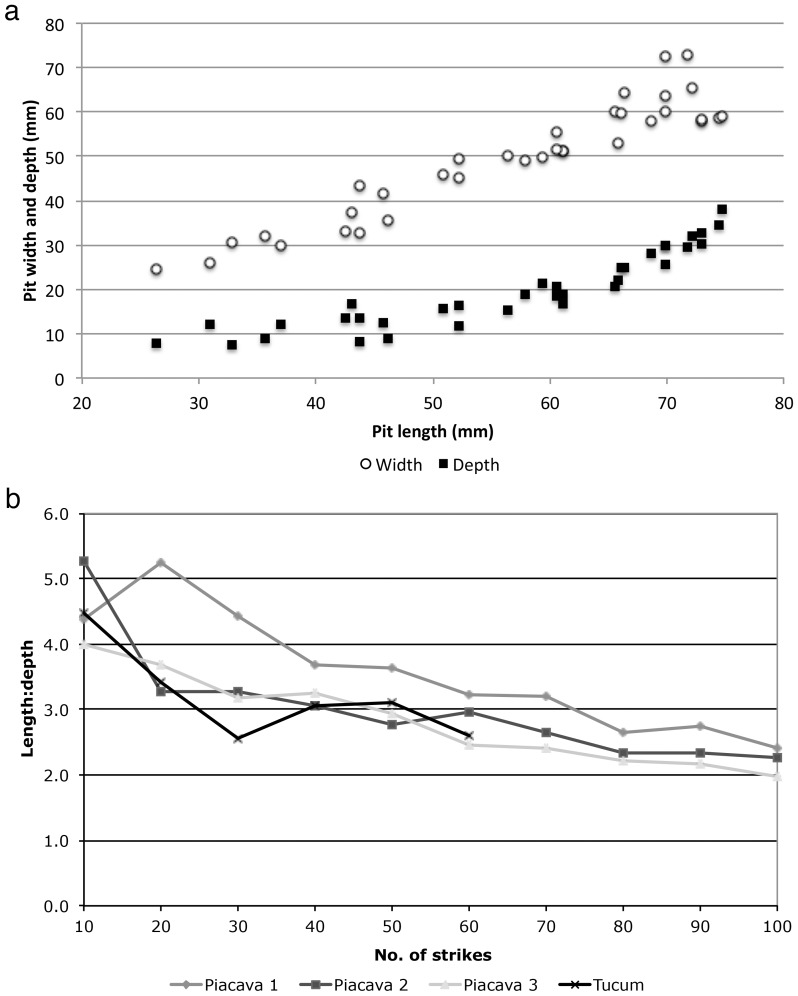
Results of the stone-drop use-wear experiment: (a) pit width and depth relative to length (piaçava and tucum results combined); (b) ratio of pit length∶depth for three piaçava nuts, and one tucum nut (the tucum nut has sixty strikes, the others 100 strikes).

Results of the stone drop experiment performed by a human are very similar to those from the BN study involving capuchins. Three pits were created while processing piaçava, and these show consistent formation rates (Figure 6a). One piaçava nut, measuring 61×40 mm, was used for this experiment (it did not crack); the final pit sizes exceed these values, likely resulting from slight nut movement at the moment of impact. The tucum nuts fractured after an average of 33 strikes for each nut, which is a much lower fracture rate than that typically seen among the monkeys [23], supporting the conservative assumption of lower energy input from the stone drop experiment. One aspect that differed between the BN and stone drop studies is that the stone drop protocol did not also involve striking nuts placed beside the pits, which explains the perhaps unrealistic greater maximum pit depth (close to 40 mm) and the high number of strikes to crack tucum attained in the latter study. Placing nuts beside the pits would likely have abraded that surface and therefore slightly increased the length and width of the pits already present, while decreasing their relative depth. Although they did not attain the same maximum size in this study, pits resulting from tucum processing were not distinguishable via measurement ratios from those created by piaçava nuts (Figure 6b).

## Discussion

Damage clearly identifiable as resulting from capuchin tool use occurred within a day of establishing the BN at FBV. This damage included rapid formation of the distinct pits seen at most anvils used by wild capuchins in the local area, along with break-up and removal of the bedded sandstone surface. Initiation of new pits was dominated by the activities of the alpha male, potentially as a result of his greater strength and body mass [26].

On the small BN anvil, the maintenance of more than five or six pits appeared difficult, as pits would start to join together or be lost through abrasion and destruction of the surrounding surface. Larger and harder anvils than the BN stone could no doubt sustain a greater number of pits, as evidenced by the anvil diversity seen across the FBV landscape [7], [16]. The published average density of pits for stone anvils measured at FBV is 6.6 pits/m^2^ (anvils average 7.8 pits and 1.89 m^2^; range 0–43 pits and 0–43.4 pits/m^2^), and anvils around the same mesa as the field laboratory average 9.5 pits/m^2^ (averages of 9.8 pits and 1.29 m^2^) [7]. BN had a maximum pit density of 39.5 pits/m^2^ (6 pits and 0.15 m^2^), at the higher end of previously recorded values, but within the natural range. The two highest pit densities published at FBV occur at the same mesa as the field laboratory (MM23: 31.1 pits/m^2^; MM30: 43.4 pits/m^2^), as does a previously unpublished and heavily-pitted anvil with a density of 16.9 pits/m^2^ (83 pits and 4.89 m^2^). These elevated densities may relate either to intensity of use or the relative softness of the sandstone in this part of the FBV site.

New pits were formed on the BN anvil with approximately every 10 nuts cracked, or 38 strikes. While these data provide an initial guide for interpreting the abundance of pits at anvils formed from similar sandstone at FBV, we caution against uncritically applying this metric to other types of stone surface (e.g. harder sandstones), to other capuchin sites, or to other primate sites. Additional factors that may mediate the formation of pits must be considered, such as weathering rates, the processing of different food types to those seen here, and the size of the anvil (the closeness of pits to the edge of small anvils most probably affects the likelihood of edge fractures affecting or removing those pits). These data also do not apply to the formation of use-damage on wooden anvils, which require separate study. The main criterion used here to measure anvil damage, percussion pitting, will be less useful in areas with more resistant anvils [27], or in circumstances where particular environments alter anvil characteristics more readily (e.g., the inter-tidal zones exploited by tool-using long-tailed macaques in Thailand [8], [28]).

The stone hammer material may also influence the rate of anvil pit formation. Specifically, use of softer rocks as hammers likely results in absorption of more of the force of a blow into the hammer itself, deforming or even breaking the hammer rather than the nut and underlying substrate. The dominant use of quartzite in this experiment precludes our testing this hypothesis, but we would expect the use of softer hammers to lengthen the time required for pit formation, rather than eliminating pit formation altogether.

Our data from the initial period of observation allow us assess the approximate rate of BN use over the subsequent three years. The initial processing of 228 nuts produced 42% of the estimated mass lost between 2011 and 2014, so we estimate that the monkeys have processed around 550 nuts in total on the BN anvil. Total strikes are estimated at over 1900 in that time. Under natural conditions (outside the initial study period), we therefore estimate 106 nuts and 373 strikes per year at BN. It is possible that BN became less appealing following its initial use as an anvil, due to its reduced size and damage, but we have no data to test this hypothesis.

The debris field of sandstone anvil fragments and broken nut shells surrounding BN is distinctive, and composed of durable materials that constitute an archaeological signature [16]. Similar debris fields surround other anvils at FBV, and reduction in anvil volume and height has been qualitatively observed among the anvils at the field laboratory since commencement of research in 2003 (Figure 1b,c). At BN, sediment levels adjacent to the anvil have seen both decreases and increases, with the most notable change being the accretion of around 4 cm sediment to the anvil's south and west. South-west is the direction of the nearby mesa, which is the main sediment source at the field laboratory.

Whole nuts were preferentially placed within existing pits, while partially cracked nuts were more often placed outside of pits. We propose that this behavior may result from (a) the fragmented partial nuts being smaller and therefore less accessible if placed within pits, (b) the rounded whole nuts being more prone to move during striking, and be lost, if not placed within a controlling pit [see 24], [26], and/or (c) partially cracked nuts being more stable when placed on a flat surface, because they are typically placed and struck with a flat facet downwards once initially broken. Neither adults nor immature monkeys consistently used existing pits as opposed to other exposed portions of the anvil surface or the most recently used pit. If pits form part of a constructed material niche [29], then we could say that the BN experiment influenced the behavior of immature and adult individuals to a similar extent in this experiment.

Adult males primarily engaged in cleaning, and in particular the current and former alpha males, although monkeys of all ages and sexes conducted this behavior. Cleaning activities continually exposed the surface of the anvil directly to impact with the processed nuts, and assisted in the concurrent build-up of sandstone and nut debris surrounding the anvil. These activities appear important therefore for the archaeological recognition of anvils as activity centers, both as surface evidence of tool use and in the formation of a subsurface material record.

Previous studies of pit formation and interpretation from primatological and paleoanthropological contexts have typically not considered the impact of anvil maintenance on wear rates and types [4]–[7], [9], which the capuchin data indicate may be a useful complement to existing research. Use-wear research also generally does not consider how demographic factors may influence damage patterns or rates, but the influence of large male capuchins as opposed to females or immature monkeys at FBV marks this as another area worthy of further investigation. For example, cleaning by alpha males may be an example of a socially-partitioned role [e.g., 30], or it may be that extra force employed by some males generates additional debris, requiring more frequent cleaning to maintain a stable nut position. The fact that one lower-ranking adult male engaged in cleaning only at low levels, similar to the adult females, does not allow us to distinguish between these options at present.

Finally, we note that some archaeological sites in northeast Brazil (e.g., in Serra da Capivara National Park) contain pitted sandstone surfaces likely resulting from human activity (MH, personal observation). These may be the outcome of unintentional anthropogenic use-damage, or they may be cupules resulting from deliberate human manufacture. Data on both the formation and shape of anvil use-damage from capuchin monkey nut-cracking activities are required to properly assess the origins of cupules or use-damaged pits found at archaeological sites in this region.
